# Membrane Cholesterol Inhibits Progesterone-Mediated Sperm Function through the Possible Involvement of ABHD2

**DOI:** 10.3390/ijms24119254

**Published:** 2023-05-25

**Authors:** Luca De Toni, Ilaria Cosci, Iva Sabovic, Andrea Di Nisio, Diego Guidolin, Federica Pedrucci, Federica Finocchi, Stefano Dall’Acqua, Carlo Foresta, Alberto Ferlin, Andrea Garolla

**Affiliations:** 1 Department of Medicine, Unit of Andrology and Reproductive Medicine, University of Padova, 35128 Padova, Italy; luca.detoni@unipd.it (L.D.T.); iva.sabovic@gmail.com (I.S.); andrea.dinisio@unipd.it (A.D.N.); federica.pedrucci@unipd.it (F.P.); federica.finocchi@unipd.it (F.F.); carlo.foresta@unipd.it (C.F.); alberto.ferlin@unipd.it (A.F.); 2 Veneto Institute of Oncology IOV-IRCCS, 35128 Padova, Italy; ilaria.cosci@yahoo.it; 3 Department of Neuroscience, Section of Anatomy, University of Padova, 35128 Padova, Italy; diego.guidolin@unipd.it; 4 Department of Pharmaceutical Science, University of Padova, 35128 Padova, Italy; stefano.dallacqua@unipd.it

**Keywords:** molecular dynamics, migration assay, cyclodextrin, LC-MS, sperm motility

## Abstract

Abhydrolase domain containing 2-acylglycerol lipase (ABHD2) was recently claimed as the membrane receptor of progesterone (P4) in sperm cells, mediating cell processes such as sperm chemotaxis and acrosome reaction. Here, we investigated the role of membrane cholesterol (Chol) on ABHD2-mediated human sperm chemotaxis. Human sperm cells were obtained from twelve normozoospemic healthy donors. ABHD2–Chol interaction was modelled by computational molecular-modelling (MM). Sperm membrane Chol content was depleted by incubating cells with cyclodextrin (CD) or augmented by the incubation with the complex between CD and Chol (CD:Chol). Cell Chol levels were quantified by liquid chromatography-mass spectrometry. Sperm migration upon P4 gradient was evaluated through the accumulation assay in a specific migration device. Motility parameters were evaluated by sperm class analyzer, whilst intracellular calcium concentration, acrosome reaction and mitochondrial membrane potential were evaluated with calcium orange, FITC-conjugated anti-CD46 antibody and JC-1 fluorescent probes, respectively. MM analysis showed the possible stable binding Chol to ABHD2, resulting in to major impact on the protein backbone flexibility. The treatment with CD was associated with a dose-dependent increase in sperm migration in a 160 nM P4 gradient, together with increase in sperm motility parameters and levels of acrosome reaction. The treatment with CD:Chol was associated with essentially opposite effects. Chol was, thus, suggested to inhibit P4-mediated sperm function through the possible inhibition of ABHD2.

## 1. Introduction

Sperm capacitation and acrosome reaction are crucial post-ejaculatory events in the fertilization process. Capacitation is a complex process occurring in spermatozoa within the female genital tract, during which sperm cells acquire a gain in progressive motility, called hypermotility, prodromal to the oocyte penetration and fertilization [1]. In this frame, key biochemical events, such as the increase in intracellular pH, the opening of sperm-specific Ca^2+^ channels and the activation of cAMP-dependent protein kinase, require a precise spatial-temporal triggering by the environmental changes that the sperm encounters during his transit through the female genital tract [2]. In turn, the correct capacitation allows the sperm cell to undergo to the acrosome reaction (AR), consisting in the rupture of the acrosome cap, upon the binding on the zona pellucida, and the release of hydrolytic enzymes functional to sperm penetration [3]. A key role in capacitation and acrosome reaction triggering is exerted by progesterone (P4), released by granulosa cells of the cumulus oophore [4]. In addition, P4 has a recognized role as chemotactic agent driving sperm motility in the nearest distance from the oocyte, according to its increasing concentration gradient [5]. In this regard, the membrane receptor mediating most of P4 activities in sperm cells was recently identified as the abhydrolase domain containing 2-acylglycerol lipase (ABHD2) [6,7]. According to the proposed P4-ABHD2-endocannabinoid axis model in sperm cells, the binding of P4 to ABHD2 would activate the lipid hydrolase function of the receptor, depleting the endocannabinoid 2-arachidonoyl glycerol (2AG) from the plasma membrane. Since 2AG exerts an inhibitory action toward the sperm-specific calcium channel called CatSper, the ABHD2-dependent removal of 2AG would remove CatSper inhibition, resulting in intracellular calcium influx, increased sperm motility and triggering of the acrosomal reaction [7]. The dependence of the sperm capacitation process on the lipid composition of the cell membrane is supported by a large amount of studies [8]. In particular, a critical factor was represented by membrane levels of cholesterol (Chol), being the reduction in the membrane cholesterol concentration, compared to the immediate post-ejaculatory levels, a necessary prerogative for the achievement of capacitation and subsequent acrosome reaction [9].

The membrane Chol content is thought to actively affect the function of the membrane proteins located therein [10,11]. However, the precise molecular mechanisms subtending these processes are still under-investigated. Because of its polycyclic, condensed and saturated structure, membrane Chol is recognized to reduce the degrees of conformational freedom of the molecular moieties immediately nearby, such as phospholipids and proteins [12]. However, more specific mechanisms of protein activity regulation by Chol are increasingly being disclosed. In a recent study from our group, we showed that the membrane Chol content in sperm cells reduced the temperature gradient-oriented motility, or thermotaxis, through the likely interaction with a Chol recognition amino acid consensus sequence of the transient receptor potential vanilloid 1 (TRPV1) ion channel [13]. Even more recently, the human voltage-gated proton channel (hHv1), whose activity is involved in sperm hypermotility gain during capacitation, was shown to be functionally inhibited by Chol through the stabilization of the voltage-sensing S4 segment at resting conformation [14].

In this study, we aimed to address the possible influence of membrane Chol content on P4 related sperm chemotaxis, as a major P4-mediated cell function, through the involvement of ABHD2 as the recognized P4 membrane receptor.

## 2. Results

### 2.1. Docking Analysis of Cholesterol on ABHD2

Based on the BLAST results and on recently reported data [6], bromoperoxidase A2 from *Streptomyces aureofaciens* (PDB code: 1BRO) was chosen as a template to model the human ABHD2 structure. The obtained model was further analyzed using the Ramachandran plot, according to which 79.6% of the residues were reported to be in the most favored regions, 17.1% in additionally allowed regions, 2.6% in generously allowed regions and only 0.7% remaining in the disallowed regions.

**Figure 1:**
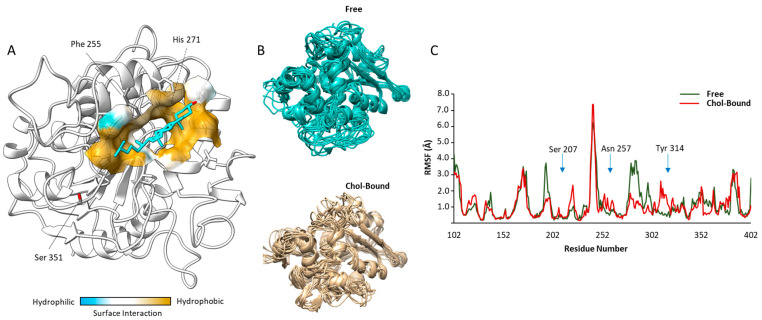
*Results of computational-based docking and molecular dynamics analysis*: (**A**) Representative image of the most energetically favorable docking solution of cholesterol (Chol, cyan) on the human model Abhydrolase Domain Containing 2- Acylglycerol Lipase (ABHD2, white). Surface modelling of inter-molecular interactions within the possible Chol binding is graphically represented as a color scale from blue (hydrophilic) to ocher (hydrophobic). (**B**) Molecular dynamics solutions of the backbone trajectories of the unliganded (Free) and Chol-bound model of ABHD2. (**C**) Root Mean Square Fluctuations (RMSF) diagrams, per amino acid position, of the Free and Chol-bound model of ABHD2. Key amino acids involved in the catalytic site (Ser 207) and substrate binding (Asn 257 and Tyr 314, respectively) are indicated.

The energetically most favorable docking region predicted for Chol corresponded to a pocket among the residues His271, Phe255 and Ser351 (Figure 1A). The estimated free Gibbs energy variation (ΔG) for the binding of Chol to ABHD2 model was, −8.3 kcal/mol, suggesting some stability of the resulting complex. However, the qualitative analysis of the Chol binding site to ABHD2 model showed that the expected inter-molecular interactions were mainly hydrophobic, with no evident formation of H-bonds between the protein and the ligand involving the hydroxy-group of Chol.

The possible influence of binding of Chol on ABHD2 function was evaluated through the variation of the protein chain flexibility with a molecular dynamic approach. Representative images of the protein backbone trajectories of free and Chol-bound ABHD2 model are reported in Figure 1B. The corresponding root mean square fluctuations (RMSF) diagrams per amino acid position are reported in Figure 1C. The major changes in the backbone flexibility were observed in the regions between 190–230 and 250–320 of the model, where differences in RMSF between the free and the Chol-bound form of the protein may reach values up to ~3.0 Å. Interestingly, in these regions are located the amino acids mainly involved in the enzyme functions [6], namely Ser^207^, a key residue of the GXSXG-motif based catalytic site of ABHD2, Asn^257^ and Tyr^314^, two polar amino acids involved in the binding with long-chain fatty acid esters.

Taken together, these data suggest that the binding of Chol to P4 membrane receptor ABHD2 is thermodynamically possible and may open the possibility of a modulation of the enzyme influencing binding to its substrates.

### 2.2. Setup of P4 Gradient Migration Test and Modification of the Sperm Membrane Cholesterol Content

**Figure 2:**
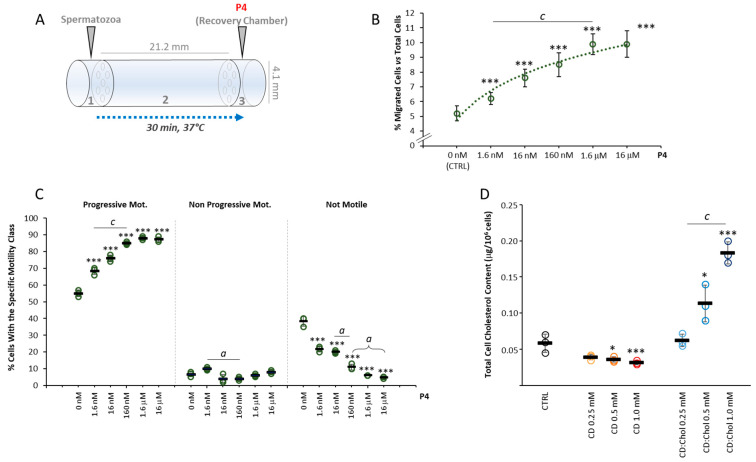
*Setup of P4 gradient migration test and modification of the sperm membrane cholesterol content*: (**A**) Representative scheme of the migration device used for the accumulation assay, depicting the size of the device and the arrangement of the sperm loading chamber (1), the migration chamber (2) and the recovery/progesterone (P4) chemoattractant chamber (3). (**B**). Correlation between the P4 concentration in the recovery chamber and the rate of sperm migration, expressed as a percentage of cells loaded the loading chamber. Significance: *** = *p* < 0.001 vs. CTRL; c = *p* < 0.001 between the indicated conditions. (**C**) Sperm motility parameters of cells migrated towards different concentrations of P4, evaluated by Sperm Class Analyzer (SCA) and reported as percentage of cells with progressive motility, non-progressive motility and not motile cells. Significance *** = *p* < 0.001 vs. CTRL; a = *p* < 0.05 and c = *p* < 0.001 between the indicated conditions. (**D**) Results of cholesterol (Chol) quantification in sperm cells, performed by liquid chromatography-mass spectrometry (LC-MS), in untreated sperm cells (CTRL) and in sperm cells incubated with cyclodextrin (CD), or CD loaded with cholesterol (CD:Chol), at concentrations of ranging from 0.25 to 1 mM, respectively. Data are expressed as μg Chol per 1 × 10^6^ sperm cells. Significance: * = *p* < 0.05, and *** = *p* < 0.001 vs. CTRL; c = *p* < 0.001 between the indicated conditions.

An initial series of experiments were performed in order to address the proper concentration of P4 to be used in subsequent migration assays, through the migration device that was built as previously described (Figure 2A) [13,15]. To this aim, a dose–response curve was created by relating the concentration of P4 as chemoattractant and the percentage of cells accumulated within the recovery chamber of the migration device after a migration period of 30 min at 37 °C, as detailed in the methods section. Cells were allowed to migrate towards the P4 concentration gradient generated by filling the recovery chamber with a P4 solution in sperm washing medium (SWM), at a concentration ranging from 0 nM (CTRL) to 16 μM. In CTRL conditions, a baseline sperm migration of less than 6% was observed, attributable to the random drift of spermatozoa (Figure 2B). As the concentration of P4 increased, a significant increase in the percentage of migrated spermatozoa was observed (respectively: 6.2 ± 0.4% P4 1.6 nM; 7.6 ± 0.6% P4 16 nM; 8.5 ± 0.8% P4 160 nM; 9.9 ± 0.7% P4 1.6 μM, 9.9 ± 0.9% P4 16 μM; all *p* < 0.001 vs. CTRL and *p* < 0.001 values among conditions between P4 1.6 nM and P4 1.6 μM). At a concentration of P4 ≥ 1.6µM, there was no additional increase in sperm accumulation in the recovery chamber, suggesting some saturating effect of the P4 gradient. The change in motility parameters of migrated spermatozoa at the different P4 concentrations tested was also evaluated (Figure 2C). Compared with cells migrated in CTRL conditions, cells migrated towards a concentration gradient of P4 showed levels of progressive motility progressively and significantly higher with the increase in the concentration of P4 generated in the recovery chamber (all *p* < 0.001 vs. CTRL). In parallel, significantly decreasing rates of not motile cells were observed with the increase in P4 concentration (*p* < 0.001 vs. CTRL and among all other conditions). For the purpose of subsequent migration tests, the highest concentration of P4 associated sperm migration was chosen, namely 160 nM.

In order to modify the cholesterol content in the sperm plasma membrane, 2-hydroxypropyl-ß-clodextrin (CD) was used as a molecular carrier of hydrophobic species, as previously described [13,16]. Incubation with di CD saturated with Chol (CD:Chol), used as Chol donor, was performed to increase the membrane Chol content as previously described [17]. This method was based on the fact that, given the small representation of cytoplasm in spermatozoa and the short reaction times, the interaction between cells and CD, as Chol, allows essentially the exchange of membrane Chol [17]. The efficiency of membrane Chol levels modification was monitored by the quantification of total Chol content by ultra-high-performance liquid chromatography associated with mass-spectrometry detector (UPLC-MS). Total Chol content was initially quantified in sperm cells incubated for 30 min with CD at concentrations ranging from 0 to 1 mM (Figure 2D). UPLC-MS analysis showed that along with the increase in CD concentration, a significant decrease in sperm Chol content of was observed (respectively: CD 0 mM, 0.066 ± 0.003 μg/10^6^ cells vs. CD 0.25 mM, 0.050 ± 0.007 μg/10^6^ cells; CD 0.5 mM, 0.049 ± 0.002 μg/10^6^ cells and CD 1.0 mM, 0.042 ± 0.002 μg/10^6^ cells; all *p* values < 0.01 vs. CD 0 mM). On the other hand, incubation with CD:Chol for 30 min, at a concentration ranging between 0 and 1 mM, showed a slight but non-significant increase in total Chol content in sperm incubated at the lowest concentration of 0.25 mM (0.067 ± 0.014 μg/10^6^ cells *p* = 0.950 vs. CD:Chol 0 mM) and a strong and significant increase in total Chol content in sperm cells incubated at higher concentrations (CD:Chol 0.5 mM 0.120 ± 0.022 μg/10^6^ cells and CD:Chol 1.0 mM 0.188 ± 0.032 μg/10^6^ cells; *p* = 0.013 and *p* < 0.001 vs. CD:Cdol 0 mM, respectively).

### 2.3. Effect of the Depletion of Cell Chol Levels on P4-Mediated Sperm Chemotaxis

**Figure 3:**
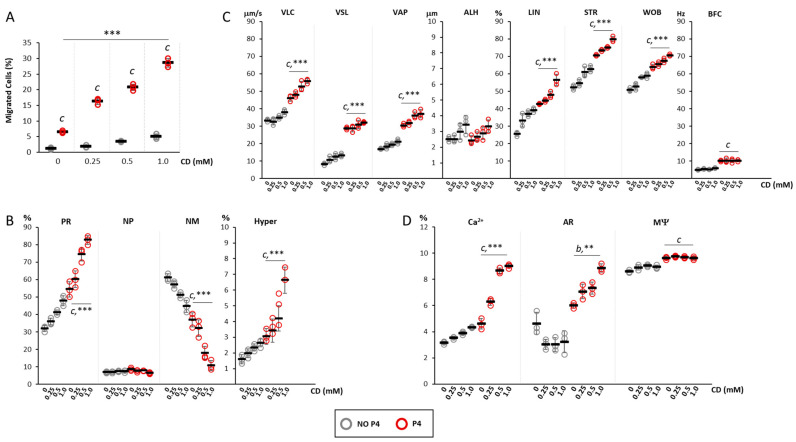
*Effect of the depletion of cell cholesterol levels on progesterone-mediated sperm chemotaxis*: (**A**) Results of the accumulation test, showing the migration of sperm cells, pre-incubated with cyclodextrin (CD) at concentrations ranging from 0 mM to 1 mM, in the absence (NO P4) or in the presence of a progesterone gradient (P4). Data are reported as the percentage of migrated cells. Significance: c = *p* <0.001 P4 vs. the corresponding NO condition P4; *** = *p* < 0.001 for cumulative effect CD+P4. (**B**) Motility classes of sperm cells retrieved in the recovery chamber upon migration, in NO P4 or P4 conditions, and incubated at different concentrations of CD. Samples were evaluated by sperm class analyzer (SCA) and classified as having progressive motility (PR), non-progressive motility (NP), no motility (NM) and hyperactive (Hyper) motility, and reported as percentages of total cells evaluated. Significance: c = *p* < 0.001 P4 vs. the corresponding NO P4 condition; *** = *p* < 0.001 for cumulative effect CD+P4. (**C**) Detailed motility parameters of sperms distinguished into curvilinear velocity (VLC), straight velocity (VSL), mean path velocity (VAP), lateral head travel amplitude (ALH), linearity (LIN), straightness (STR as VSL/VAP), wobble (WOB as VAP/VLC) and cross beat frequency (BCF). Significance: c = *p* < 0.001 P4 vs. the corresponding condition NO P4; *** = *p* < 0.001 for cumulative effect CD+P4. (**D**) Analysis of intracellular calcium levels, acrosome reaction and mitochondrial function, obtained by fluorescence microscopy and flow cytometry, respectively. Data are reported as percentages of calcium orange positive cells (Ca^2+^) cells, CD46-positive cells (AR) cells, and JC1-positive (MΨ) cells. Significance: b = *p* < 0.01 and c = *p* < 0.001 P4 vs. the corresponding condition NO P4; ** = *p* < 0.01 and *** = *p* < 0.001 for cumulative effect CD+P4.

On the bases of observed reduction in cell Chol content by CD treatment, the effect of membrane Chol depletion on P4-mediated sperm chemotaxis was assessed by incubating spermatozoa selected by swim-up for 30 min with CD, at concentrations ranging from 0 to 1 mM, and then quantifying sperm accumulation in the recovery chamber of the migration device after 30 min migration at 37 °C in absence (NO P4) and in presence of a 160 nM P4 gradient (P4, Figure 3A). In NO P4 conditions, sperm migration was generally less than 2% and the incubation with CD was associated some non-significant increase in the percentage of migrated cells, regardless of CD concentration. The application of a P4 gradient, as expected, was associated with a highly significant increase in cells accumulated in the recovery chamber. In addition, the extent of the migration was further increased by the incubation with CD in a CD concentration-dependent manner (respectively: CD 0 mM, 6.48 ± 0.38%; CD 0.25 mM, 16.3 ± 0.99%; CD 0.5 mM 20.77 ± 1.05%, 28.63 ± 1.4% CD 1 mM, respectively; *p* < 0.001 P4 vs. NO P4 for all conditions, *p* < 0.001 for cumulative CD+P4 effect).

Spermatozoa recovered in the recovery chamber were also evaluated for motility parameters (Figure 3B). Compared with cells migrated in NO P4 conditions, cells migrated towards a P4 gradient had a significantly higher percentage of spermatozoa with progressive motility (CD 0 mM, *p* < 0.001 P4 vs. NO P4), hyperactivated cells (CD 0 mM, *p* < 0.005 P4 vs. NO P4) and a significantly lower level of not motile spermatozoa (CD 0 mM, *p* < 0.001 P4 vs. NO P4). The incubation with CD at a concentration ranging from 0.25 to 1.0 mM was associated with a further concentration-dependent increase in the percentages of cells with progressive motility (*p* < 0.005 for cumulative CD+P4 effect) and with hyperactivated motility (*p* < 0.001 for cumulative CD+P4 effect). An opposite trend was observed for not motile cells (*p* < 0.001 for cumulative CD+P4 effect). Detailed motility parameters obtained by CASA analysis (Figure 3C) showed that, compared with NO P4 conditions, cells migrated towards a P4 gradient had significantly higher values of VLC, VSL, VAP, LIN, STR, WOB, BCF (*p* < 0.001 P4 vs. NO P4). The incubation with CD followed by migration toward the P4 gradient was associated with a further CD dose-dependent increase in the parameters VLC, VAP, LIN, STR, WOB (*p* < 0.05 for cumulative CD+P4 effect).

Finally, the effects on the functional status of sperm cells in terms of percentages of cells with increased intracellular calcium content (Ca^2+^), cells that underwent acrosomal reaction (AR) and cells with correct mitochondrial potential (MΨ) were evaluated (Figure 3D). Migration towards the P4 gradient was generally associated with the increase in cells with high intracellular calcium content compared with the NO P4 conditions (*p* < 0.001 P4 vs. NO P4). Incubation with CD, at a concentration ranging from 0.25 to 1.0 mM, was associated with a significant increase in this cell population which was dependent on the CD concentration- (*p* < 0.001 for cumulative CD+P4 effect). Additionally, the migration toward the P4 gradient showed no significant variation of cells that underwent AR, compared to NO P4 conditions, in absence of CD (*p* > 0.05 P4 vs. NO P4). Differently, the incubation with CD was associated with a significant and dose dependent percentage of cells showing AR (*p* < 0.01 for cumulative CD+P4 effect). Finally, a slight but significant increase in cells with high MΨ was observed in sperm that migrated towards the P4 gradient compared with NO P4 conditions (*p* < 0.001 P4 vs. NO P4). However, incubation with CD, regardless of the concentration tested, was associated with no variation of this parameter in both conditions.

### 2.4. Effect of the Increase in Cell Chol Levels on P4-Mediated Sperm Chemotaxis

**Figure 4:**
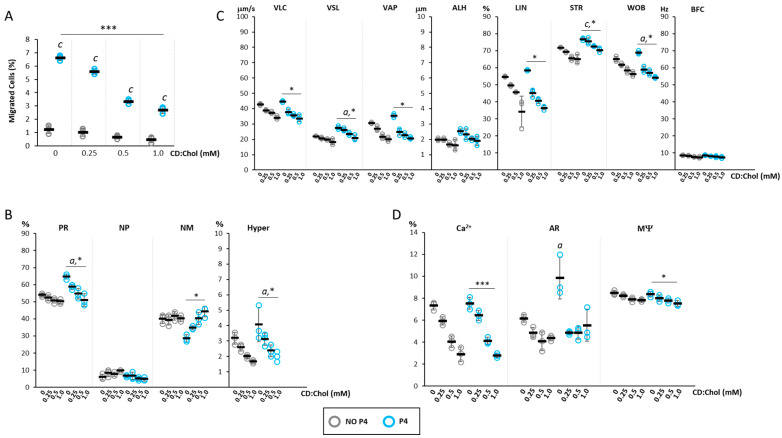
*Effect of the increase in cell cholesterol levels on progesterone-mediated sperm chemotaxi*: (**A**) Results of the accumulation test, showing the migration of sperm cells, pre-incubated with or CD loaded with cholesterol (CD:Chol) at concentrations ranging from 0 mM to 1 mM, in the absence (NO P4) or in the presence of a progesterone gradient (P4). Data are reported as the percentage of migrated cells. Significance: c = *p* <0.001 P4 vs. the corresponding NO condition P4; *** = *p* < 0.001 for cumulative effect CD+P4. (**B**) Motility classes of sperm cells retrieved in the recovery chamber upon migration, in NO P4 or P4 conditions, and incubated at different concentrations of CD:Chol. Samples were evaluated by sperm class analyzer (SCA) and classified as having progressive motility (PR), non-progressive motility (NP), no motility (NM) and hyperactive (Hyper) motility, and reported as percentages of total cells evaluated. Significance: a = *p* < 0.05 P4 vs. the corresponding NO P4 condition; * = *p* < 0.05 for cumulative effect CD+P4. (**C**) Detailed motility parameters of sperms distinguished into curvilinear velocity (VLC), straight velocity (VSL), mean path velocity (VAP), lateral head travel amplitude (ALH), linearity (LIN), straightness (STR as VSL/VAP), wobble (WOB as VAP/VLC) and cross beat frequency (BCF). Significance: a = *p* < 0.05 and c = *p* < 0.001 P4 vs. the corresponding condition NO P4; * = *p* < 0.05 for cumulative effect CD+P4. (**D**) Analysis of intracellular calcium levels, acrosome reaction and mitochondrial function, obtained by fluorescence microscopy and flow cytometry, respectively. Data are reported as percentages of calcium orange positive cells (Ca^2+^) cells, CD46-positive cells (AR) cells, and JC1-positive (MΨ) cells. Significance: a = *p* < 0.05 P4 vs. the corresponding condition NO P4; * = *p* < 0.05 and *** = *p* < 0.001 for cumulative effect CD+P4.

On the bases of observed cell Chol increase by CD:Chol treatment, the effect of membrane Chol increase on P4-mediated sperm chemotaxis was assessed by incubating swim-up selected spermatozoa for 30 min with CD:Chol, at concentrations ranging from 0 to 1 mM, and then quantifying sperm accumulation in the recovery chamber of the migration device after 30 min migration at 37 °C in NO P4 conditions or in the presence of a 160 nM P4 gradient (Figure 4A).

The basal low migratory activity observed in NO P4 conditions showed a further lowering trend after incubation with CD:Chol, despite being non-significant. As expected, the basal application of a P4 gradient showed a significant increase in cell migration (*p* < 0.001 P4 vs. NO P4). However, incubation with CD:Chol was associated with a significant reduction in the percentage of cell migration, the extent of which was closely associated with the concentration of the CD:Chol complex (respectively, CD:Chol 0 mM 6.62 ± 0.2%; CD:Chol 0.25 mM 5.6 ± 0.2%; CD:Chol 0.5 mM 3.34 ± 0.19%; CD:Chol 1 mM 2.7 ± 0.27%; *p* < 0.001 for cumulative CD:Chol+P4 effect).

Spermatozoa recovered in the recovery chamber were also evaluated for motility parameters (Figure 4B), showing the reduction in the percentage of cells with progressive and hyperactivated motility in a CD:Chol concentration-dependent manner (*p* < 0.05 P4 vs. NO P4; *p* < 0.05 for cumulative CD:CHOL+P4 effect). Consistently, the fraction of immotile spermatozoa showed a significant increasing trend (*p* < 0.05 for cumulative effect CD:Chol+P4).

The evaluation of detailed sperm motility parameters, assessed by the CASA system (Figure 4C), showed that the cells that migrated toward the P4 gradient, compared with those migrated in NO P4 conditions, had higher values of VSL, WOB (*p* < 0.05 P4 vs. NO P4) and STR (*p* < 0.001 P4 vs. NO P4). However, incubation with CD:Chol was associated with a general and concentration-dependent reduction in these parameters in migrated spermatozoa in either NO P4 and P4 conditions. Specifically, a highly significant reduction was observed for the parameters VLC, VSL, VAP, LIN, STR and WOB (*p* < 0.05 for cumulative CD:CHOL+P4 effect).

Finally, the effects on the functional status of sperm cells in terms of Ca^2+^, AR and MΨ were evaluated (Figure 4D). The incubation with CD:Chol was associated with a significant decrease in the percentage of cells with high levels of Ca^2+^ in both cells migrated towards a P4 gradient and in NO P4 conditions (*p* < 0.001 for cumulative CD:Chol+P4 effect; *p* < 0.001 for cumulative CD:Chol+NO P4 effect). On the other hand, the percentage of cells undergone to AR showed to be not significantly affected by the incubation with CD:Chol in NO P4 condition. Differently, cells undergone to AR upon P4 migration at basal (CD:Chol 0 mM) were significantly increased compared to the corresponding NO P4 condition. Incubation with CD:Chol was associated with a significant blunt of AR regardless of the concentration of the latter. The percentage of cells with high MΨ also showed a significant CD:Chol concentration-dependent reduction in either cell migrated in a NO P4 conditions and towards a P4 gradient (*p* < 0.05 for cumulative CD:CHOL+P4 effect; *p* < 0.05 for cumulative CD:CHOL+NO P4 effect).

## 3. Discussion

In this study, we provide evidence of a widened role of membrane cholesterol levels in human sperm cells function, being negatively associated with progesterone-mediated effects, along with the previously highlighted temperature-driven migration [13], in a concentration-dependent manner. In addition, a possible direct effect of Chol on the enzyme function of the P4 membrane receptor ABHD2 was suggested by a computational approach.

Sterols, and in particular cholesterol (Chol), are key components of the plasma membrane, where they modify major biophysical properties such as the phospholipid bilayer fluidity and lateral diffusion of integral lipids and proteins [18]. Particularly in spermatozoa, membrane Chol was long recognized in the regulation of cell motility and capacitation [19]. Accordingly, functional variations of sperm, from the early phases from spermatogenesis to the fertilization process, are generally related with changes of Chol levels in sperm function [10,11]. It is a common opinion that Chol acts more specifically than just modifying membrane rigidity. However, the molecular targets of Chol activity are currently under intense investigation. In this regard, we recently showed that membrane Chol behaves as a functional inhibitor of TRPV1, a transient receptor potential channel involved in sperm thermotaxis, namely the ability of cell to migrate towards and increasing temperature gradient [13,20]. Most importantly, membrane Chol inhibits TRPV1 through the likely specific interaction with the putative cholesterol recognition amino acid consensus (CRAC) sequence in the S5 helix domain, as demonstrated by the lower potency of epi-cholesterol: the 3-hydroxy epimer of Chol showing a sub-optimal inter-molecular interaction with the putative binding site on TRPV1 [13]. More recently, another molecular target of Chol was identified as the human voltage-gated proton (hHv1), an acid extruder channel involved in the regulation of intracellular pH of many cell types, including spermatozoa [21]. Han et al. showed that Chol stabilizes the voltage-sensing S4 domain of hHv1 channel in a resting conformation supporting the link between Chol reduction and cytoplasmic alkalization, a triggering event for calcium influx through the CatSper calcium channel and, in turn, for the motility gain [14]. Of note, CatSper is also a downstream effector of the P4 membrane receptor ABHD2 in spermatozoa, since its activation cleaves 2-arachidonoyl glycerol, which is a CatSper inhibitor [7].

In this study, we provided evidence of a further checkpoint of sperm function regulated by membrane Chol, namely P4-mediated sperm activation. Indeed, we showed that membrane Chol levels, experimentally modified by the use of CD as sterol carriers, are linearly and inversely correlated with all the recognized P4-mediated functions in spermatozoa: chemotaxis, intracellular calcium concentration, motility gain and acrosome reaction. In addition, through a computational approach, we showed that these effects are possibly exerted by the direct interaction between Chol and ABHD2. In fact, on a structural model of ABDH2 protein, we described a possible docking solution Chol for which the binding is thermodynamically favored. In addition, the molecular dynamic analysis suggested that the binding of Chol has minor effects on the backbone flexibility of the ABHD2 catalytic site, but rather on conformational freedom of key amino-acids involved in the binding of the enzyme substrates [6]. Unfortunately, we were not able to provide any additional cues on the molecular details of this interaction model, since neither a crystallographic structure of ABHD2 nor a known P4 antagonist on this receptor are currently available. In this regard, biolayer interferometry analysis will be helpful in support of the computational findings [22].

However, the working hypothesis here proposed sounds likely, since no additional or alternative P4 receptor on sperm cell was identified so far. Further studies are required to address the molecular basis of the binding of P4 to ABHD2 and the modulation of its hydrolase activity.

## 4. Materials and Methods

### 4.1. Chemicals

Cholesterol, progesterone (P4), 2-hydroxypropyl-ß-cyclodextrin (CD) and Superclean TM LC-Si SPE Tube 1 mL (Supleco, Bellefonte, PA, USA) were all purchased from Merck Life Science S.r.l. (Milano, Italy). Sperm washing medium (SWM) SWM was purchased from FUJIFILM Europe (Tilburg, The Netherlands). The fluorescent probes Calcium Orange™ and 5′,6,6′-tetrachloro-1,1′,3,3′-tetraethylbenzimidazolylcarbocyanine iodide (JC-1) were purchased from Thermo Fisher Scientific (Milan, Italy). Mouse monoclonal fluorescein isocyanate (FITC)-conjugated anti-human CD46 antibody was purchased from BD-Biosciences (Milan, Italy).

### 4.2. Semen Samples

Each patient provided written informed consent for participation in the study, which had been previously authorized by the local Ethical Committee for Clinical Trials of the Padova University Hospital (protocol n. 2208P and subsequent amendments). The investigation was performed according to the principles of the Declaration of Helsinki. Semen was obtained from twelve healthy normozoospermic volunteers (aged 30–40 years), according to the criteria of the World Health Organization, 10th edition (WHO 2010). Exclusion criteria were no medical history for previous genital tract infections, cryptorchidism, testicular torsion, or varicocele.

Semen collection was by masturbation in a sterile container after 2–5 days of sexual abstinence. Following collection, all samples were allowed to liquefy for 30 min at 37 °C. All specimens had normal viscosity and appearance. The sperm concentration of each specimen was measured by Makler^®^ counting chamber (Santa Ana, CA, USA) and phase-contrast microscope (Nikon Eclipse 50i) at 20× magnification. WHO parameters related to sperm motility were evaluated using the computerized Sperm Class Analyzer (SCA, Microptic S.L, Barcelona, Spain). Briefly, a 10-μL aliquot of each sample was placed in a Makler^®^ counting chamber and analyzed by a phase-contrast microscope connected to the computer presenting the Sperm Class Analyzer (SCA) software (Hamilton Thorne, Beverly, MA, USA). The measurements performed included: progressive motility (PR: sperm actively moving, in either linear or in a wide circle, regardless of speed), non-progressive motility (NP: sperm with all other motility patterns with an absence of progression, e.g., swimming in small circles) and motionless (IM: no movement). Kinetic parameters involved: the amplitude of lateral head displacement (ALH, µm), curvilinear velocity (VCL, µm/s), straight-line velocity (VSL, µm/s), average path velocity (VAP, µm/s), linearity (LIN = VSL/VCL, %), wobble (WOB = VAP/VCL, %) and straightness (STR = VSL/VAP, %).

### 4.3. Docking Study

The amino acid sequence of ABHD2 was obtained from the Uniprot database (P08910) (https://www.uniprot.org/, accessed on 1 April 2023). Homology modeling was performed by using the protocol available on the web server SWISS-MODEL (https://swissmodel.expasy.org, accessed on 1 April 2023). The modelling pipeline involves the search in a library for suitable templates using BLAST and HHblits (see [23] for references). When the template search was complete, templates and alignments were first filtered to remove redundancy and they were then ranked according to expected quality of the resulting models, as estimated by global model quality estimate (GMQE) [23]. Models were then generated by using the ProMod3 [24] modelling engine and estimates on expected accuracy of each model were obtained by the QMEAN scoring function [25]. After a final check of its stereo-chemical quality using PROCHECK software (https://www.ebi.ac.uk/thornton-srv/software/PROCHECK/, accessed on 1 April 2023) [26], the model obtaining the best score was selected for further processing.

The molecular structure of cholesterol was obtained from the PubChem database [27]. Receptor and ligand models were then prepared for docking using the Dock prep module available in the UCSF Chimera package [28]. This procedure involved adding hydrogen, electrostatic charges and assigning binding orders. The molecules and proteins thus drawn were then made to interact using the docking protocol provided by the Swissdock web service (http://www.swissdock.ch, accessed on 1 April 2023). The computational pipeline was based on the docking software EADock (http://www.swissdock.ch, accessed on 1 April 2023) [29]. First, the whole target protein structure was considered (blind docking) and 10,000 predicted binding modes were generated. Simultaneously, their CHARMM energies were estimated [29]. Then, solutions with the most favorable energies were ranked, taking account of the solvent effect using the FACTS implicit solvation model, and clustered [30]. The energetically most favorable binding mode was considered as the final solution.

Molecular dynamics procedure, based on the CABSflex method by Kuriata et al. [31] and available as a web server (https://biocomp.chem.uw.edu.pl/CABSflex2, accessed on 1 April 2023), was used to evaluate the conformations that the receptor can acquire after binding with Chol and to compare them with those of the free receptor. The CABS method employed coarse-grained representation of a polypeptide chain and the solvent effect was accounted for in an implicit fashion. The dynamics of CABS-based coarse-grained proteins was simulated by a random series of local conformational transitions (controlled by a Monte Carlo method and modulated by the model force field). The adopted protocol settings were those indicated by Jamroz et al. [32]. With these settings, indeed, the coarse-grained method was demonstrated to generate a similar picture of protein flexibility compared to NMR ensembles [33] and to all-atom 10–20 ns MD runs performed with explicit water at constant pressure (1013.25 hPa) and temperature (300 K) using standard force fields (see [32] for details).

### 4.4. Membrane Cholesterol Modifications

The Chol content of sperm cell membrane was modified with CD or Chol-loaded CD (CD:Chol), as previously described [13,34,35,36]. Briefly, after preliminary semen evaluation, the sample volume was divided into 4 equal aliquots, 3 of which were intended for treatments and 1 for control. All semen aliquots were washed once with SWM by centrifugation at 2500× *g* rpm for 10 min to separate seminal plasma from the cells. In Chol depletion experiments, sperm cell pellets were incubated for 30 min at 37 °C with CD solutions in SWM at the concentration of 0.25 mM, 0.5 mM or 1 mM, respectively. In Chol addition experiments, Chol was delivered to sperm membrane through the incubation for 30 min at 37 °C with the water soluble complex CD (CD:Chol) at the concentration of 0.25 mM, 0.5 mM or 1 mM, respectively. CD:Chol complex was prepared as previously described by Purdy and Graham with slight modifications [37]. In controls samples, cells were resuspended in 1 mL of SWM, with the addition of no other reagent. All sperm cell suspensions, at an almost consistent number of 50.0 ± 3.8 × 10^6^ cells, were incubated with CD or CD:Chol for 30 min at room temperature and were then washed with SWM to remove the excess. Cells destined to accumulation assays were diluted in 500 µL of fresh SWM, whereas samples for cholesterol quantification were washed twice in phosphate-buffered saline (PBS), centrifuged and pellets were stored at −80 °C until use.

### 4.5. Lipid Extraction and Cholesterol Quantification

The total lipid fraction was extracted with a 1:1 chloroform–methanol mixture from the sperm cells pellet, as previously described [38]. After resuspension in 200 μL of chloroform, the sterol fraction was isolated from lyophilized extracts by solid-phase extraction (SPE) on a 1-mL silica column, eluted with acetone and lyophilized in a Vacufuge^®^-Concentrator plus. The eluate was dissolved in methanol and 20 μL were analyzed by reverse-phase (RP) liquid-chromatography coupled to high-resolution mass spectrometry (LC-MS). A Poroshell 120 EC-C18 column (4.6 mm × 150 mm, 2.7-μm particle size) from Phenomenex (Torrance, CA, USA) was equilibrated and eluted at 40 °C with an acetonitrile/methanol solution (65:35 *v*/*v*), at a flow rate 0.3 mL/min using an Agilent (Santa Clara, CA, USA) 1290 Infinity Ultra-High Performance Liquid Chromatography (UPLC) system, equipped with a 920 autosampler and connected to a Waters (Milford, MA, USA) Xevo-G2S Q-TOF mass spectrometer, as previously described [13].

### 4.6. Sperm Migration Assay

Sperm migration assay was performed by exposing sperm cells to a progesterone (P4) gradient in a device specially designed for chemo-separation. A 38 mm length tube was used, divided internally into three compartments: two chambers at the ends, each 4 mm in diameter, respectively, one for loading and one for retrieving migrated cells corresponding to the beginning and end point of sperm migration, respectively; the 3 cm-long sector allowed intermediate translocation. For the detection of sperm responses to P4-induced chemoattraction, each sperm cell sample was placed in the loading chamber. A P4 solution in SWM at a final concentration ranging from 0 to 16 μM (500 μL) was placed in the recovery chamber. The intermediate compartment was filled with fresh SWM to create a bridge between the two chambers. The P4 solution let diffuse toward the loading chamber for a standard time of 5 min, so as to establish a P4 gradient along the bridge. Sperm cells were then migrated along the gradient to the recovery chamber for 30 min at 37 °C. As negative controls, all migration tests were repeated in the absence of attractive P4 gradient, where the recovery chamber was filled with 500 μL of SWM, and named NO P4 conditions.

### 4.7. Analysis of Sperm Parameters

The analysis of sperm parameters was evaluated as previously described [13]. Briefly, the percentage of sperm cells with high intracellular calcium levels were estimated by staining with the long-wavelength calcium indicator Calcium Orange™ (Thermo Fisher). Cells undergone to acrosome reaction were assessed by flow cytometry using the FITC-labelled mouse anti-human CD46 antibody as previously described. The percentage of cells with elevated trans-membrane potential of mitochondria (MΨ) was evaluated by the fluorescent lipophilic cationic dye JC-1 that differentially labels mitochondria according to low or high MΨ when excited at 488 nm, as previously described [39].

### 4.8. Statistical Analysis

Statistical analysis of data was performed with SPSS 21.0 for Windows (SPSS, Chicago, IL, USA). Data are expressed as means ± standard deviation (SD) and report the mean values of three independent experiments. The Kolmogorov–Smirnov test was used to verify the normal distribution of continuous data. Variables not showing normal distribution were log transformed. Multivariate analysis of variance was performed to test for differences in measured parameters between groups. Main effects included treatment (free or Chol-loaded CD), P4 gradient and their interaction; post hoc analysis was performed to test differences between presence and absence of P4 gradient at each tested CD concentration with Bonferroni correction for multiple comparisons. *p* values < 0.05 were considered as statistically significant.

## 5. Conclusions

In conclusion, our data suggested a regulatory role of membrane Chol on sperm chemotaxis through the possible inhibition of ABHD2, the recognized membrane receptor of P4 on sperm cells. Further studies are required to ascertain the molecular bases of the possible interaction between P4 and ABHD2.

## Data Availability

Data will be made available upon request.
